# Supplementary material for: Breast cancer-related lymphedema and recurrence of breast cancer: Protocol for a prospective cohort study in China
Source: PLoS One. 2023 May 16;18(5):e0285772. doi: 10.1371/journal.pone.0285772 (PMC10187897; doi:10.1371/journal.pone.0285772)
Supplement: Attachment — Submitted filename: Lighlighted for Corrections.pdf [file pone.0285772.s001.pdf]

PLOS ONE

Breast cancer-related lymphedema and recurrence of breast cancer: Protocol for a prospective cohort study in China

--Manuscript Draft--

**Manuscript Number:**	PONE-D-22-27177
**Article Type:**	Study Protocol
**Full Title:**	Breast cancer-related lymphedema and recurrence of breast cancer: Protocol for a prospective cohort study in China
**Short Title:**	Breast cancer-related lymphedema and recurrence of breast cancer: Protocol for a prospective cohort study
**Corresponding Author:**	Shaoyong Wu<br>West China Hospital of Medicine: Sichuan University West China Hospital<br>Chengdu, Sichuan CHINA
**Keywords:**	breast cancer-related lymphedema (BCRL); recurrence of breast cancer; depression
**Abstract:**	Introduction<br>The primary aim is to determine the factors associated with breast cancer-related lymphedema,<br>and to identify new associated factors for recurrence of breast cancer and depression. The secondary aim is to investigate the incidence of breast cancer-related events (breast cancer-related lymphedema, recurrence of breast cancer and depression). Finally, we want to explore and validate the complex relationship among a myriad of factors influencing breast cancer complications and recurrence of breast cancer.<br>Methods<br>A co[illegible] study of females with unilateral breast cancer will be conducted in West China Hospital between February 2023 and February 2026. Breast cancer survivors aged 17-55 years will be recruited before breast cancer surgery. We will recruit 1557 preoperative patients with a first invasive breast cancer diagnosis. Consenting breast cancer survivors will complete demographic information, clinicopathological factors, sur information, baseline information and a baseline depression questionnaire. Data were collected at four stages: the perioperative stage, chemotherapy therapy stage, radiation therapy stage, and follow-up stage. Data including the incidence and correlation of breast cancer-related lymphedema, recurrence of breast cancer, depression and medical cost will be collected and computed through the four stages above. For every statistical analysis, the participants were classified into two groups based on whether they developed secondary lymphedema. Incidence rates of breast cancer recurrence and depression were calculated separately for groups. Multivariate logistic regression will be used to determine whether secondary lymphedema and other parameters have the ability to predict recurrence of breast cancer.<br>Discussion<br>Our prospective cohort study will contribute to establish the early detection programme for breast cancer-related lymphedema and recurrence of breast cancer which both are associated with poor quality of life and reduced life expectancy. Our study also can provide new insights into the physical, economic, treatment-related and mental burdens of breast cancer survivors.
**Order of Authors:**	Linli Zhuang
	Qian Chen
	Huaying Chen
	Xuemei Zheng
	Shaoyong Wu
	Xia Liu
	Zhenzhen Feng
	Li Liu

Powered by Editorial Manager® and ProduXion Manager® from Aries Systems Corporation

	Xiaolin Shen
**Additional Information:**	
**Question**	**Response**
**Financial Disclosure**<br><br>Enter a financial disclosure statement that describes the sources of funding for the work included in this submission. Review the submission guidelines for detailed requirements. View published research articles from *PLOS ONE* for specific examples.<br><br>This statement is required for submission and **will appear in the published article** if the submission is accepted. Please make sure it is accurate.<br><br>**Unfunded studies**<br>Enter: *The author(s) received no specific funding for this work.*<br><br>**Funded studies**<br>Enter a statement with the following details:<br>• Initials of the authors who received each award<br>• Grant numbers awarded to each author<br>• The full name of each funder<br>• URL of each funder website<br>• Have or will the sponsors or funders play any role in the study design, data collection (when applicable) and analysis, decision to publish, or preparation of the manuscript?<br>• **NO** - Include this sentence at the end of your statement: *The funders had and will not have a role in study design, data collection and analysis, decision to publish, or preparation of the manuscript.*<br>• **YES** - Specify the role(s) played.<br><br>* typeset	This work was supported by the Sichuan Science and Technology Support Program (2015SZ017). The funders had and will not have a role in study design, data collection and analysis, decision to publish, or preparation of the manuscript.
**Competing Interests**<br><br>Use the instructions below to enter a competing interest statement for this submission. On behalf of all authors, disclose any competing interests that could be perceived to bias this work—acknowledging all financial support and any other relevant financial or non-financial competing interests.	NO authors have competing interests

This statement is required for submission and **will appear in the published article** if the submission is accepted. Please make sure it is accurate and that any funding sources listed in your Funding Information later in the submission form are also declared in your Financial Disclosure statement.<br><br>View published research articles from *PLOS ONE* for specific examples.<br><br>**NO authors have competing interests**<br><br>Enter: *The authors have declared that no competing interests exist.*<br><br>**Authors with competing interests**<br><br>Enter competing interest details beginning with this statement:<br><br>*I have read the journal's policy and the authors of this manuscript have the following competing interests: [insert competing interests here]*<br><br>* typeset	
**Ethics Statement**<br><br>Enter an ethics statement for this submission. This statement is required if the study involved:<br><br>• Human participants<br>• Human specimens or tissue<br>• Vertebrate animals or cephalopods<br>• Vertebrate embryos or tissues<br>• Field research<br><br>Write "N/A" if the submission does not require an ethics statement.<br><br>General guidance is provided below. Consult the submission guidelines for detailed instructions. **Make sure that all**	The study was approved by the Ethics Committee of West China Hospital (Approval No. of ethics committee: 2019 Annual Review No. 664). Registration number: ChiCTR1900025534 (Name of the registry: Chinese Clinical Trial Registry (ChiCTR) http://www.chictr.org.cn/)

Powered by Editorial Manager® and ProduXion Manager® from Aries Systems Corporation

information entered here is included in the Methods section of the manuscript.<br><br>**Format for specific study types**<br><br>**Human Subject Research (involving human participants and/or tissue)**<br>• Give the name of the institutional review board or ethics committee that approved the study<br>• Include the approval number and/or a statement indicating approval of this research<br>• Indicate the form of consent obtained (written/oral) or the reason that consent was not obtained (e.g. the data were analyzed anonymously)<br><br>**Animal Research (involving vertebrate animals, embryos or tissues)**<br>• Provide the name of the Institutional Animal Care and Use Committee (IACUC) or other relevant ethics board that reviewed the study protocol, and indicate whether they approved this research or granted a formal waiver of ethical approval<br>• Include an approval number if one was obtained<br>• If the study involved *non-human primates*, add *additional details* about animal welfare and steps taken to ameliorate suffering<br>• If anesthesia, euthanasia, or any kind of animal sacrifice is part of the study, include briefly which substances and/or methods were applied<br><br>**Field Research**<br><br>Include the following details if this study involves the collection of plant, animal, or other materials from a natural setting:<br>• Field permit number<br>• Name of the institution or relevant body that granted permission	
**Data Availability**<br><br>Authors are required to make the data underlying their research findings fully available, without restriction.	N/A - the protocol does not report results

If the manuscript reports pilot or preliminary results, the data underlying those results must be made available at the time of publication. A Data Availability Statement describing where the data can be found is required at submission. Your answers to this question constitute the Data Availability Statement and will be published in the article, if accepted. When legal or ethical requirements prohibit public data sharing, authors must indicate how researchers may obtain access to the data. See the PLOS Data Policy and FAQ for detailed information.<br><br>**Important:** Stating 'data available on request from the author' is not sufficient. If your data are only available upon request, select 'No' for the first question and explain your situation in the text box.<br><br>For protocols without pilot or preliminary data, authors are strongly encouraged to state how they plan to share research data from their study when it is completed or published.<br><br>Do the authors confirm that EITHER the protocol does not report results OR all data underlying the preliminary results will be made fully available without restriction?	
**Describe where the data may be found in full sentences. If you are copying our sample text, replace any instances of XXX with the appropriate details.**<br><br>• If **no pilot data are reported** in the manuscript, please enter the following: *No datasets were generated or analysed during the current study. All relevant data from this study will be made available upon study completion.*<br>• If the manuscript reports pilot data **held in a public repository**, include URLs, accession numbers or DOIs. If this	Deidentified research data will be made publicly available when the study is completed and published.

information will only be available after acceptance, indicate this by ticking the box below. For example: *All XXX files are available from the XXX database (accession number(s) XXX, XXX.).*<br>• If the manuscript reports pilot data contained **within the manuscript and/or Supporting Information files**, enter the following:<br>*All relevant data are within the manuscript and its Supporting Information files.*<br>• If neither of these applies but you are able to provide **details of access elsewhere**, with or without limitations, please do so. For example:<br><br>*Data cannot be shared publicly because of [XXX]. Data are available from the XXX Institutional Data Access / Ethics Committee (contact via XXX) for researchers who meet the criteria for access to confidential data.*<br><br>*The data underlying the results presented in the study are available from (include the name of the third party and contact information or URL).*<br>• This text is appropriate if the data are owned by a third party and authors do not have permission to share the data.<br><br>For study protocols with data management or sharing plans, authors are encouraged to briefly describe in their Data Availability Statement how data they generate will be made accessible when the study is completed. For example:<br>• Deidentified research data will be made publicly available when the study is completed and published.<br><br>* typeset	
Additional data availability information:	

Manuscript

Click here to access/download;Manuscript;main Manuscript.docx

Breast cancer-related lymphedema and recurrence of breast cancer:

Protocol for a prospective cohort study in China

Linli Zhuang,[1] Qian Chen,[2] Huaying Chen,[3] Xuemei Zheng,[3] Xia Liu,[4] Zhenzhen Feng,[3,5] Shaoyong Wu,[3] Li Liu[3], Xiaolin Shen[1*]

E-mail: 1637194326@qq.com

Address for correspondence: Department of Head and Neck Oncology, Cancer Center, West China Hospital, Sichuan University, No. 37 Guo Xue Alley, Chengdu, Sichuan, China.

Abstract

Introduction

The primary aim is to determine the factors associated with breast cancer-related lymphedema, and to identify new associated factors for recurrence of breast cancer and depression. The secondary aim is to investigate the incidence of breast cancer-related events (breast cancer-related lymphedema, recurrence of breast cancer and depression). Finally, we want to explore and validate the complex relationship among a myriad of factors influencing breast cancer complications and recurrence of breast cancer.

Methods

A cohort study of females with unilateral breast cancer will be conducted in West China Hospital between February 2023 and February 2026. Breast cancer survivors aged 17-55 years will be recruited before breast cancer surgery. We will recruit 1557 preoperative patients with a first invasive breast cancer diagnosis. Consenting breast cancer survivors will complete demographic information, clinicopathological factors, sur information, baseline information and a

baseline depression questionnaire. Data were collected at four stages: the perioperative stage, chemotherapy therapy stage, radiation therapy stage, and follow-up stage. Data including the incidence and correlation of breast cancer-related lymphedema, recurrence of breast cancer, depression and medical cost will be collected and computed through the four stages above. For every statistical analysis, the participants were classified into two groups based on whether they developed secondary lymphedema. Incidence rates of breast cancer recurrence and depression were calculated separately for groups. Multivariate logistic regression will be used to determine whether secondary lymphedema and other parameters have the ability to predict recurrence of breast cancer.

Discussion

Our prospective cohort study will contribute to establish the early detection programme for breast cancer-related lymphedema and recurrence of breast cancer which both are associated with poor quality of life and reduced life expectancy. Our study also can provide new insights into the physical, economic, treatment-related and mental burdens of breast cancer survivors.

would decrease physical and mental function, increase disease burden and increase financial costs. It is a common complication in breast cancer patients. Considering the establishment of a baseline of upper limb and tracking changes, a screening cohort of secondary lymphedema may be the optimal solution to develop screening programs and evaluate the prevalence of breast cancer-related lymphedema in China.

Breast cancer recurrence (BCR) is not curative and a hammer blow for breast cancer survivors[3]. It directly threatens breast cancer survivors' life expectancy and decreases their quality of life. BCRL and BCR share risk factors such as higher BMI, pathological type, breast cancer location, Tumor Node Metastasis(TNM) staging and comorbidities and treatment-related factors (surgery, radiotherapy and chemotherapy)[4]. Early detection helps implement personal and precise medical precautions for patients with secondary lymphedema (or breast cancer recurrence).

Indeed, up to 30% of breast cancer survivors had depressive symptoms[5]. Depression is associated with an increased disease burden and treatment-related cost and a decreased ability to cope with breast cancer. Fear of cancer recurrence also affects mental health, especially anxiety and depression, directly[6]. Given this situation, our study also implements depressive symptom screening in breast cancer survivors.

A previous study found that recurrence of breast cancer may have a negative impact on breast cancer-related lymphedema. However, whether BCRL is associated with the risk of recurrence of breast cancer is uncertain. This study aimed to investigate and evaluate the incidence and correlation of BCRL, BCR and depression. In addition, the disease burden of breast cancer is also an important area of concern. It is a multidimensional construct encompassion surgery,

oncology-related treatment, medicine costs and depression, and other elements destroying everyday life. A full screening plan in breast cancer may catch cancer early and improve the healthy situation and prognosis.

Methods

Project context

This study is a prospective cohort study in breast cancer survivors. Based on the retrospective data of the electronic medical record system in West China Hospital, we found a 3-year BCRL incidence rate of 17.57% after breast surgery or breast reconstruction in patients with breast cancer. The rate of BCR occurrence was 15.53% at three years. For a more specific answer to influence factors and correlation for BCRL, BCR and depression in breast cancer survivors, we will have to recruit patients with breast cancer before they receive breast cancer-related treatment in a prospective study design and continue to evaluate the disease burden of breast cancer. The main outcomes include the incidence and correlation of BCRL and BCR within 3 years. This time frame is chosen because 3 years is a high-incidence period of BCRL and BCR. The secondary outcome is depression. Our study period is between February 2023 and February 2026. This study was approved by the Ethics Committee of West China Hospital (Approval No. of ethics committee: 2019 Annual Review No. 664). Registration number: ChiCTR1900025534 (Name of the registry: Chinese Clinical Trial Registry (ChiCTR) http://www.chictr.org.cn/). Figure 1 shows participant flow.

Study sample

We will conduct a prospective cohort study of females with unilateral breast cancer in West China Hospital. We will recruit breast cancer patients aged 18-55 years who will receive unilateral

radical mastectomy or modified radical mastectomy, postoperative chemotherapy and radiotherapy. Patients with breast cancer will be first diagnosed in West China Hospital, and surgery and treatment data will be retrieved from the electronic medical record system. This cohort study will commence one week prior to breast cancer surgery and conclude after the whole 3-year follow-up period. .

Our study will need 1557 preoperative patients with a first-time invasive breast cancer diagnosis. Patients must meet the following criteria for inclusion.

- Female subjects who were between 18 and 55 years old.
- Understand and sign the informed consent.
- Recruited in West China Hospital.
- First diagnosis of invasive breast cancer.
- Patients must not be treated with neoadjuvant radiotherapy before.
- Breast cancer patients will receive either radical or modified radical mastectomy.

There are four exclusion criteria; they are described briefly below.

- Patients with bilateral breast cancer
- Patients could not complete all the treatment and 3-year follow-up.
- There was significant asymmetry in the size of the upper extremities.
- The patients had cardiac dysfunction or renal and hepatic insufficiency.

Study procedure

Baseline assessment

Basic demographic and treatment information will be collected through an electronic medical record system. Our study will gather self-reported demographic information, including age, sex,

marital status, education level, etc. We will obtain surgery and treatment information, including surgery date, surgery type (axillary lymph node dissection or sentinel lymph node dissection), total number of dissected lymph nodes, chemotherapy regimen, radiotherapy regimen and cycle number of treatment (chemotherapy/radiotherapy).

Ongoing assessment

Table 1 shows the standard assessment process of the follow-up assessment of our study. Clinical evaluations will include assessment of BCRL, RBC and depression. At each follow-up visit, patients will receive upper-limb volume assessment and physical and imaging examination[7]. The research nurse will help them complete the Beck Depression Inventory II questionnaire about patients' mental status.

Diagnosis standards

Diagnosis of BCRL: We used a 200 ml limb volume difference as the diagnostic standard. The 200 ml limb volume difference standard is a universal criterion recommended by the 2020 Consensus Document of the International Society of Lymphology[3].

Diagnosis of RBC: Physical examination and magnetic resonance imaging (MRI) for screening surveillance of breast cancer survivors. Breast cancer survivors who had biopsy-proven recurrence of breast cancer at presentation documented by fine-needle aspiration biopsy or core needle biopsy[8-12].

Diagnosis of depression: The screening tool will be the Beck Depression Inventory II. We used the Beck Depression Inventory II rubric; 0 to 4 was no depressive symptoms, 5 to 7 was mild, 8 to 15 was moderate, and 16 to more was severe[13-14].

Record

We will record the outcomes in detail, including the onset time, diagnosis time, symptoms, adherence, and medicine cost. The incidence in each stage will be calculated and used to explore complex relationships among the outcomes.

Data collection

Data will be collected at four stages: the perioperative stage, chemotherapy therapy stage, radiation therapy stage, and follow-up stage. Having completed the previous stage, we move on to the next. The total healthcare expenditure for each treatment period will be computed. All authors could access information that could identify individual participants during data collection and statistical analysis.

First stage-- perioperative stage

Data will be collected at the time of the perioperative stage, including demographic data (sex, age, race, ethnicity, marital status, socioeconomic status, educational status and BMI), clinicopathological factors (pathological type, breast cancer location, TNM staging and comorbidities), surgery information (date of diagnosis, date of surgery, type of surgery, type of surgical incision, lymph node management, the total number of dissected lymph nodes), and baseline information (upper-limb volume difference, scale results of Beck Depression Inventory II). Patient information in the perioperative stage will be collected through the electronic medical record after obtaining written informed consent. We will manage to build a follow-up database preoperatively. We will use this follow-up database for baseline and data collection, transfer and storage.

Secondary stage-- chemotherapy therapy stage

Data will be collected at the time of the chemotherapy therapy stage, including the number of

chemotherapy cycles, body mass index (BMI), upper limb volume measurement, assessment of tumor recurrence, and scale results of Beck Depression Inventory II. Once they receive the first round of chemotherapy, participants will be followed up in a regular oncology ward each cycle. Data collectors will conduct breast cancer-related lymphedema surveillance through upper-limb volume measurements. For patients who increase the upper-limb volume difference by more than 200 ml, secondary lymphedema is confirmed and diagnosed by a physician and physical therapist. Data collected at the time of the radiation therapy stage included the total number of radiation therapy cycles, body mass index (BMI), upper limb volume measurement, assessment of tumor recurrence, and scale results of the Beck Depression Inventory II.

Third stage-- radiation therapy stage

Radiation therapy cycles terminate at relatively short times. Therefore, data on the main factors will only be collected before radiation therapy cycles and after stopping the planned radiation treatment.

Last stage-- follow-up stage

The follow-up stage is also the last stage of our cohort study. After finishing radiation and chemotherapy treatment, patients will be followed until 3 years after breast cancer surgery. Postoperative breast cancer patients are exposed to a chance of recurrence. The implementation of guideline-recommended follow-up plans for breast cancer patients is necessary. The patients in our study will be seen for follow-up visits once every three months[16]. Data collected at the time of the follow-up stage included BMI, upper limb volume measurement, assessment of tumor recurrence, and scale results of the Beck Depression Inventory II. Detailed cohort protocols in the four research stages are presented in Table 2.

Quality assurance

To ensure the uniformity of data, all the nurses in our study will undergo unified training about the measurement of arm volume and how to provide guidance when patients complete the questionnaire. Arm volume will be measured twice, in the morning and at night, and the average of two measurements will be used in the analyses to minimize errors. All oncologists will receive unified training, including magnetic resonance imaging (MRI) analysis and diagnosis of breast cancer recurrence. Moreover, loss to follow-up will continue to hinder data completeness of our cohort study. Therefore, the computer and application are programmed to alert patients by telephone and message before one day when breast cancer patients need to complete the follow-up.

Statistical analyses

The following main factors will be calculated when each of the study stages (perioperative stage, chemotherapy therapy stage, radiation therapy therapy stage, follow-up stage) is completed: BCRL incidence, RBC rate, and incidence of depression. To calculate the incidence of the primary outcomes, we will sum the total of participants within each of the stages as the denominator and summed the total of people with one of the primary outcomes as the numerator. For every statistical analysis, the participants will be classified into two groups based on whether they developed secondary lymphedema. Incidence rates of breast cancer recurrence and depression were calculated separately for groups. Chi-square test will be used in comparison of rate between two groups[15]. As a secondary outcome, depression symptoms will be repeatedly measured in this study. The results of the assessment of depression symptoms will be compared across periods and between treatment-related events using statistical analysis with repeated measures analysis of

variance (ANOVA).

We will identify clinicopathological factors, surgery information, lymph node management, breast cancer-related treatment and body mass index(BMI) as risk factors due to their reported associations with secondary lymphedema, recurrence of breast cancer and depression symptoms. Multivariate logistic regression will be used to determine whether secondary lymphedema and other parameters have the ability to predict recurrence of breast cancer. To assess the correlates with these risk factors and outcomes, we will describe the strength of the association using clinically meaningful and statistically significant effect sizes, and a correction will be based on demographic characteristics.

Sample size calculation

Sample size will be estimated using the sample size estimation formula for prevalence studies. ($N = \frac{\mu_{\frac{\alpha}{2}}^{2}(1-p)p}{d^{2}}$)[16-17]. The formal required sample size calculation was based on the actual incidence of the main outcomes in the electronic medical record system of West China Hospital. The maximum permissible error is 2%. Based on the retrospective data of the electronic medical record system from 2017 to 2020 in West China Hospital, we found a 3-year BCRL incidence rate of 17.57% after breast surgery or breast reconstruction in patients with breast cancer. The rate of BCR occurrence was 15.53% at three years. Assuming a 10% dropout rate, at least 1557 participants should be recruited in our cohort study. We are trying to recruit more breast cancer patients, considering the longevity of the study.

Missing data

We will assess missing data and record the study stage and reasons for missing data. Data in the previous stage will be retained and calculated if patients are lost to follow-up at this stage of

the study. Missing data will not be handled using estimation.

Patient and Public Involvement

No patient was involved.

Discussion

The final outcomes of our cohort study will include the following: (1) deep analysis of breast cancer-related lymphedema to establish the screening program of secondary lymphedema. (2) deep analysis of recurrence of breast cancer to estimate the incidence of recurrence of breast cancer. (3) Continuous assessment of depression during the perioperative, treatment and follow-up periods. (4) whole-course analysis of the incidence rate of secondary lymphedema, recurrence of breast cancer, depression and calculation hospital and treatment costs. (5) Whether breast cancer survivors with secondary lymphedema have a higher risk of breast cancer recurrence remains unknown. Our study is an explorative study. We would like to know whether early screening of secondary lymphedema could be an important addition to improving early detection and early treatment of recurrence of breast cancer. (6) The development of rich related data of breast cancer, including personal characteristics, clinicopathological factors, surgical factors, treatment factors, and financial and mental burdens, can lead to the assessment of the impacts of breast cancer on breast cancer survivors. On review of the literature, there is not previous longitudinal cohort which give serious consideration to all these six points which related to breast cancer survivors. However, these factors have direct and indirect effects on health. Therefore, all the participants in our study would benefit from a timely screening program. The results of this study will inform the establishment of a package plan for the early screening of breast cancer survivors.

Among patients with breast cancer treated, we found that being diagnosed with breast

cancer-related lymphedema is associated with a modestly increased risk of recurrence of breast cancer[18]. In fact, breast cancer-related lymphedema greatly impacts quality of life. Secondary lymphedema, as a major complication, causes the most disability in breast cancer survivors[19]. Cancer recurrence threatens patients' lives. Cancer recurrence drives the most deaths from breast cancer. According to evidence-based data, the presence of new-onset lymphedema should be confirmed to determine whether breast cancer first recurs. Moreover, conservative management is becoming the treatment of choice for secondary lymphedema. However, complete decongestive therapy (first choice of conservative management) will not be used if the neoplasm is the cause of breast cancer-related lymphedema. Early screening and identification of secondary lymphedema and recurrence of breast cancer are of enormous significance to breast cancer medical prognosis. However, there is no continuous assessment longitudinal cohort established in China. We will focus on the correlation assessment between secondary lymphedema and breast cancer recurrence. This work will be the cornerstone of the well-directed screening programme and help to develop well-defined intervention. In the current era of heightened whole-course management, serious complication screening and recurrence identification of breast cancer survivors may be even more important.

We will also focus on the economic and mental burdens of breast cancer[21]. It is associated with poor physical function, role function, emotional function, cognitive function and social function. This is because depression magnifies the possibility of negative outcomes and spends more cost and time on breast cancer-related treatment. In fact, there is no mandated screening program for depression in breast cancer survivors in China. It is unknown whether breast cancer-related treatment and medicine costs correlate with an increased risk for depression. As a

result, longer follow-up studies of depression and medicine-related costs can provide more information about the long-term economic and mental burden of breast cancer survivors. Moreover, financial stress is well established as an important reason for depression. The hospital and treatment expenses of breast cancer patients are the slowly draining income of patients and their families. Fear of breast cancer recurrence and financial stress may produce the negative effect of making the person more depressive[22].

Limitations

Data from breast cancer patients will only be collected from a large district general hospital. It is difficult to put the scheme of further multicentric trials into practice under the normalization of epidemic prevention and control. Moreover, similar to other cohort studies, the situation in which breast cancer survivors are lost to follow-up seems inevitable. The completeness of treatment and follow-up data will be important to assess the correlation between breast cancer-related lymphedema and recurrence and disease burden (depression and medicine cost). Loss to follow-up will continue to hinder data completeness of our cohort study.

Abbreviations

BCRL:Breast cancer-related lymphedema; BCR:Breast cancer recurrence; BMI:body mass index; TNM:Tumor Node Metastasis;

MRI:magnetic resonance imaging; ANOVA: analysis of variance.

Declarations

Ethics approval and consent to participate

The study was approved by the Ethics Committee of West China Hospital (Approval No. of

ethics committee: 2019 Annual Review No. 664). Registration number: ChiCTR1900025534 (Name of the registry: Chinese Clinical Trial Registry (ChiCTR) http://www.chictr.org.cn/)

Our cohort study is in line with the international ethical principles and ethical process of West China Hospital. All participants provided written informed consent. Breast cancer survivors will be informed that their identifying information will be confidential before they participate in our cohort study. We need to obtain full informed consent because our study will span almost 3 years. Participants can stop their participation at any time during the study, without disclosing reasons for withdrawing.

Consent for publication

No application.

Availability of data and materials

The data should remain confidential. Only qualified researchers and data analysts can access raw data in our study. All the demographic data will be deidentified by allocating numbers to all the patients. The datasets used during the study are available on reasonable request by contacting the corresponding author. Dissemination activities will include peer-reviewed publications and international conferences.

Competing interests

The authors declare that they have no competing interests.

Funding

This work was supported by the Sichuan Science and Technology Support Program (2015SZ017). This funding source had no role in the design of this study and will not have any role during its execution, analyses, interpretation of the data, or decision to submit results.

Authors' Contributions

L.Z., Q.C., S.W., and X.S. conceived and designed the study. and X.Z., Z.F. and L.L. contributed to the extraction, statistical analysis, and data interpretation. L.Z. and X.S. drafted the original manuscript. X.Z., L.X. and H.C. revised the intellectual content of this article. All the authors have read and approved the final version of the manuscript.

Author details

[1]Department of Rheumatology and Immunology, West China Hospital of Sichuan University, Chengdu, Sichuan, China.[2]Center of Biostatistics, Design, Measurement and Evaluation, Department of Clinical Research Management, West China Hospital of Sichuan University, Chengdu, Sichuan, China, 610041.[3]Department of Head and Neck Oncology, Cancer Center, West China Hospital, Sichuan University, Chengdu, China[4]Department of Abdominal Oncology, Cancer Center, West China Hospital, Sichuan University, Chengdu, China[5]Unit of Cancer Day Care, West China Hospital, Sichuan University, Chengdu, China

Acknowledgments

The authors thank for the support of the fund, thanks for the help of everybody in our study groups.

Supplements

All the ethical and funding approval documentations have been uploaded as Study Protocol Proof. We received governmental funding and the study protocol has undergone peer-reviewed by the funding bodies.

Our study is a proposed studies that have not completed participant recruitment at the time of submission. No publications containing the results of this study have already been published or

submitted to any journal.

Reference

1. Bundred N, Foden P, Todd C, et al. Increases in arm volume predict lymphoedema and quality of life deficits after axillary surgery: a prospective cohort study. Br J Cancer. 2020;123(1):17-25. doi:10.1038/s41416-020-0844-4
2. Executive Committee of the International Society of Lymphology. The diagnosis and treatment of peripheral lymphedema: 2020 Consensus Document of the International Society of Lymphology. Lymphology. 2020;53(1):3-19.
3. Marinac CR, Nelson SH, Breen CI, et al. Prolonged Nightly Fasting and Breast Cancer Prognosis. JAMA Oncol. 2016;2(8):1049-1055. doi:10.1001/jamaoncol.2016.0164
4. Rockson SG, Keeley V, Kilbreath S, et al. Cancer-associated secondary lymphoedema. Nat Rev Dis Primers. 2019;5(1):22. Published 2019 Mar 28. doi:10.1038/s41572-019-0072-5
5. Hahn EE, Munoz-Plaza CE, Pounds D, et al. Effect of a Community-Based Medical Oncology Depression Screening Program on Behavioral Health Referrals Among Patients With Breast Cancer: A Randomized Clinical Trial. JAMA. 2022;327(1):41-49. doi:10.1001/jama.2021.22596
6. Hall DL, Jimenez RB, Perez GK, et al. Fear of Cancer Recurrence: A Model Examination of Physical Symptoms, Emotional Distress, and Health Behavior Change. J Oncol Pract.
7. Kataja V, Castiglione M; ESMO Guidelines Working Group. Primary breast cancer: ESMO clinical recommendations for diagnosis, treatment and follow-up. Ann Oncol. 2009;20 Suppl 4:10-14. doi:10.1093/annonc/mdp114
8. Clarke M, Cutter D, Darby S, et al. Relevance of breast cancer hormone receptors and other factors to the efficacy of adjuvant tamoxifen: patient-level meta-analysis of randomized trials. Lancet. 2011

Aug 27;378(9793):771-84. doi: 10.1016/S0140-6736(11)60993-8. Epub 2011 Jul 28. PMID: 21802721; PMCID: PMC3163848.

9. Shen Songjie, Sun Qiang, Huang Xin, Li Yan, [illegible]uechong, Niu Zihan, Song Yu, Wang Yingjiao, Zhou Xingtong. Guidelines for Breast Cancer Screening in Chinese Women (2022 Edition)[J].China Research Hospital,2022,9(02):6-13.DOI:10.19450/j.cnki.jcrh.2022.02.003.

10. Gennari A, André F, Barrios CH, et [illegible]l. ESMO Guidelines Committee. Electronic address: clinicalguidelines@esmo.org. ESMO Clinical Practice Guideline for the diagnosis, staging and treatment of patients with metastatic breast cancer. Ann Oncol. 2021 Dec;32(12):1475-1495. doi: 10.1016/j.annonc.2021.09.019. Epub 2021 Oct 19. PMID: 34678411.

11. Gradishar WJ, Anderson BO, Abraham J, et al. Breast Cancer, Version 3.2020, NCCN Clinical Practice Guidelines in Oncology. J Natl Compr Canc Netw. 2020 Apr;18(4):452-478. doi: 10.6004/jnccn.2020.0016. PMID: 32259783.

12. Kataja V, Castiglione M; ESMO Guidelines Working Group. Primary breast cancer: ESMO clinical recommendations for diagnosis, treatment and follow-up. Ann Oncol. 2009 May;20 Suppl 4:10-4. doi: 10.1093/annonc/mdp114. PMID: 19454421.

13. Wang YP, Gorenstein C. Psychometric properties of the Beck Depression Inventory-II: a comprehensive review. Braz J Psychiatry. 2013 Oct-Dec;35(4):416-31. doi: 10.1590/1516-4446-2012-1048. Epub 2013 Dec 23. PMID: 24402217.

14. Iltis AS,Misra S,Dunn LB, et al. Addressing risks to advance mental health research. JAMA Psychiatry. 2013;70 (12):1363-71. doi:10.1001/jamapsychiatry.2013.2105

15. Yuan T, Fitzpatrick T,Ko NY, et al. Circumcision to prevent HIV and other sexually transmitted infections in men who have sex with men: a systematic review and meta-analysis of global data.

Lancet Glob Health. 2019;7 (4):e436-e447. doi:10.1016/S2214-109X(18)30567-9

16. Naing L, Winn T, Rusli B N. Practical issues in calculating the sample size for prevalence studies[J]. Archives of Orofacial Sciences, 2006, 1(1).

17. Bavindra A, Belavendra A, Sushil K. Sample size estimation in pravalence studies. Indian J Pediatr. 2012,79(11):1482-1488.

18. Byun H K, Chang J, Choi S H, et al. Risk of Lymphedema Following Contemporary Treatment for Breast Cancer: An Analysis of 7,426 Consecutive Patients from a Multidisciplinary Perspective. International Journal of Radiation Oncology Biology Physics, 2019, 105(1):E52-.

19. Mcduff S, Mina An I, Brunelle C L, et al. Timing of Lymphedema Following Treatment for Breast Cancer: When Are Patients Most At-Risk? International journal of radiation oncology, biology, physics, 2018, 103(1):62-70.

20. Kerlikowske, Karla, Yu-Ru Su, et, al. Association of Screening With Digital Breast Tomosynthesis vs Digital Mammography With Risk of Interval Invasive and Advanced Breast Cancer. JAMA: The Journal of the American Medical Association 2022.327(22): 2220-230. Web.Kerlikowske K, Su YR, Sprague BL, Tosteson ANA, Buist DSM, Onega T, Henderson LM, Alsheik N, Bissell MCS, O'Meara ES, Lee CI, Miglioretti DL. Association of Screening With Digital Breast Tomosynthesis vs Digital Mammography With Risk of Interval Invasive and Advanced Breast Cancer. JAMA. 2022 Jun 14;327(22):2220-2230. doi: 10.1001/jama.2022.7672. PMID: 35699706; PMCID: PMC9198754.

21. Hahn, Erin E, Corrine E Munoz-Plaza, Dana Pounds, et, al. Effect of a Community-Based Medical Oncology Depression Screening Program on Behavioral Health Referrals Among Patients With Breast Cancer: A Randomized Clinical Trial. JAMA: The Journal of the American Medical

Association 2022.327(1): 41-49. Web.

22. Ford JS,Chou JF,Sklar CA, et al. Psychosocial Outcomes in Adult Survivors of Retinoblastoma. J Clin Oncol. 2015;33 (31):3608-14. doi:10.1200/JCO.2014.60.5733

Figure

Click here to access/download;Figure;Fig 1. CONSORT 2010 flow diagram.docx

Fig 1. CONSORT 2010 flow diagram

CONSORT

TRANSPARENT REPORTING of TRIALS

CONSORT 2010 Flow Diagram

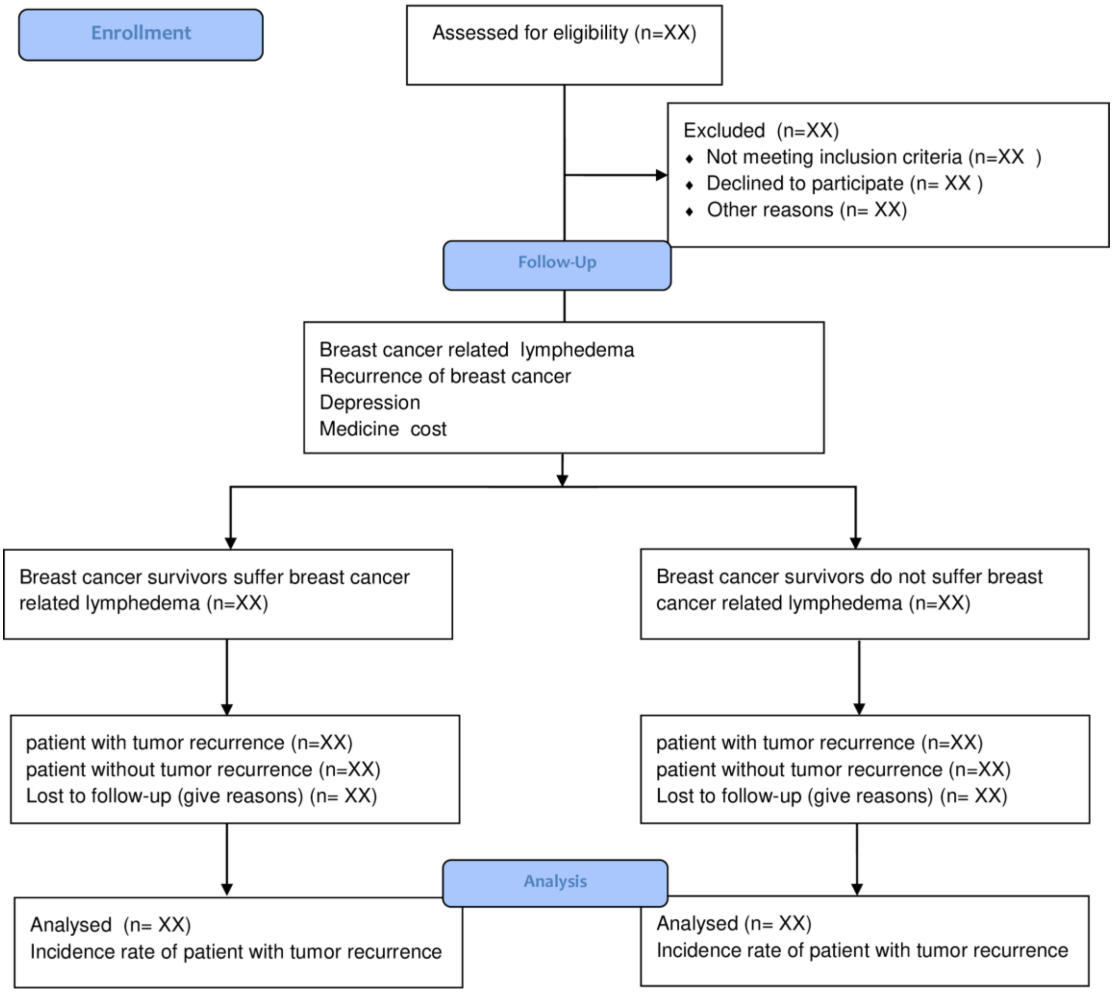

Click here to access/download;Figure;Table 1.pdf

Table 1. Standard assessment process in cohort study—timepoint and main outcomes

Time point	BMI	Patients with secondary lymphedema or not	Patients with oncology recurrence or not	Beck Depression Inventory II	medicine cost
**perioperative stage**					
before surgery	√	/	/	√	√
**chemotherapy therapy stage**					
circle 1	√	√	√	√	√
circle 2	√	√	√	√	√
circle 3	√	√	√	√	√
circle(4.5.6…n-1)	√	√	√	√	√
circle(n)—last circle	√	√	√	√	√
**radiation therapy stage**					
before radiation therapy	√	√	√	√	√
after radiation therapy	√	√	√	√	√
**follow-up stage**					
9th month(after surgery)	√	√	√	√	√
1st year(after surgery)	√	√	√	√	√
15th month(after surgery)	√	√	√	√	√
18th month(after surgery)	√	√	√	√	√
21th month(after surgery)	√	√	√	√	√
2nd years(after surgery)	√	√	√	√	√
27th month(after surgery)	√	√	√	√	√
30th month(after surgery)	√	√	√	√	√
33th month(after surgery)	√	√	√	√	√
3rd year(after surgery)	√	√	√	√	√

Table 2 Detailed cohort protocols in four research stages

**perioperative stage**	
demographic data	gender, age, race, ethnicity, marital status, socioeconomic status, educational status and body mass index (BMI)
clinicopathological factors	pathological type, breast cancer location, TNM staging and comorbidities
surgery information	date of diagnosis, date of surgery, type of surgery, type of surgical incision, Lymph node management, the total number of dissected lymph nodes
baseline information	arm circumferences, scale results of Beck Depression Inventory II
financial information	treatment related cost
**chemotherapy therapy stage**	
timepoint information	the number of chemotherapy cycles
main information	body mass index (BMI), arm volume measurement(secondary lymphedema), assessment of tumor recurrence, scale results of Beck Depression Inventory II
financial information	treatment related cost
**radiation therapy stage**	
timepoint information	total number of radiation therapy cycles
main information	body mass index (BMI), arm volume measurement (secondary lymphedema), assessment of tumor recurrence, scale results of Beck Depression Inventory II
financial information	treatment related cost
**follow-up stage**	
timepoint information	the number of follow-up cycles
main information	body mass index (BMI), arm volume measurement (secondary lymphedema), assessment of tumor recurrence, scale results of Beck Depression Inventory II
financial information	treatment related cost

Click here to access/download
Supporting Information
Original approval document from ethics committee.pdf

Click here to access/download
Supporting Information
Translated version of the approval ethics document.pdf
